# Syrian medical students’ acceptance of peer physical examination and its associating factors: a cross-sectional study

**DOI:** 10.1186/s12909-022-03985-5

**Published:** 2022-12-28

**Authors:** Jameel Soqia, Mohamad Ashraf Shamaa, Dima Alhomsi, Laila Yakoub-Agha, Mhd Basheer Alameer, Rawan Alhomsi, Mohmad Nour Hakok, Rim Khalil, Mazen Zaitouna

**Affiliations:** 1 Faculty of Medicine, Damascus University, Damascus, Syria; 2 UMR1195, University Paris Sud, INSERM, University Paris-Saclay, Le Kremlin-Bicetre, France; 3 Urology Department, Grand Hospital of Est Francilien, Meaux, France

**Keywords:** Peer Physical Examination, Syria, Middle East, PPE, Medical Education, COVID-19

## Abstract

**Background:**

This study aimed to evaluate the acceptance of Peer Physical Examination (PPE) in middle Eastern society with its associate factors, and PPE acceptance during Covid-19 pandemic. The acceptance of PPE is considered high in multiple studies carried out in the west, but there were nearly no studies investigating the acceptance of PPE in the middle east or low-income countries.

**Methods:**

A questionnaire was shared through social media with students with focus on clinical-year students. A total number of 657 medical students were collected with a 74.5% response rate. The questionnaire gathered demographic information and recorded previous experience of PPE. A 5-point-likert scale was used to assess acceptance of PPE, factors affecting it, and the influence of COVID-19 pandemic. It also considered body’s areas students would accept to be examined. Associations between participants’ demographic and other details were tested using independent-samples T Test and other tests, and a *p*-value of < 0.05 was considered significant.

**Results:**

Eighty percent of medical students accepted PPE, while 3% did not, and 17% were neutral. Males had statistically significantly higher acceptance rates of PPE (M = 3.94 out of 5). Also, females had lower acceptance of being examined by other gender than males but did not mind examining other gender. Furthermore, the groin area (thigh) was the most rejected area for examination (20% only accept it), followed by the breast (23%).

There was no statistically significant difference between different Universities groups or between different academic performance groups, finally there was statistically significant effect of religion and society on acceptance of PPE and religion has affected females more than males (*p*- value = 0.002).

70.8% of students supported PPE during the COVID-19 pandemic, while 6.8% did not, and 22.4% were neutral. There was not a significant difference in acceptance and supporting of PPE during the COVID-19 pandemic between males and females.

**Conclusions:**

With an 80% acceptance rate, PPE represents an effective alternative to the absent life models in Syrian universities. The application of PPE is less likely to go without difficulties, but authors suggest the presence of a supervisor and single-gender groups with friends paired together if possible.

**Supplementary Information:**

The online version contains supplementary material available at 10.1186/s12909-022-03985-5.

## Background

Clinical training represents a vital part of medical school. The enhancement of clinical skills is one of the main goals of any medical student. Developing an adequate set of clinical skills is important to ensure providing the best clinical care possible. There are many ways to develop clinical skills including Peer Physical Examination (PPE). PPE is an experiential way of learning anatomy or clinical skills where students learn by practicing physical examination techniques on each other [1, 2]. PPE is an important learning method as it enables medical students to become familiar with clinical and communication skills before encountering real patients [3]. It helps them build up their skills, giving them more time to practice and limiting the potential harm caused to patients [4]. PPE, therefore, offers students the opportunity to learn in an environment where they can make mistakes and learn from them [5]. It helps them learn what is ‘normal’ by Examining healthy students before examining patients with abnormal signs [6].

The main barrier preventing the routine application of PPE is the acceptance of students of varying background and religions due to what PPE causes of discomfort or potential inappropriate behavior from other students [3].

The acceptance of PPE is considered high in multiple studies carried out in the west with an acceptance rate of 98% [3], 97% [7], 78% [5] and 60% [8] but there were nearly no studies investigating the acceptance of PPE in the middle east or low-income countries.

The application of PPE is important in low-income countries generally and in Syria especially where the late conflict caused a shortage in clinical training assets, and it was later exacerbated by the spread of COVID-19 causing a weakness in clinical training.

We conducted this study in Syria, a country with a middle eastern society of many religions and ethnicities.

Our study aims to:

1. Identify the attitudes of medical students towards peer physical examination (PPE) as part of learning clinical skills.
2. Explore the relationships between students’ demographic characteristics (religion, gender, ethnicity....etc.) and their attitude toward PPE.
3. Investigate whether the COVID-19 pandemic is a barrier to applicate PPE in Syrian medical schools or not.

## Methods

### Participants and setting

This cross-sectional study was carried out in 2021 among all medical students in their first to the sixth year of study at different universities of Syria (12 public and private medical colleges) as well as graduated students. Our study mainly targeted third-year students who had just encountered clinical subjects and clinical-year students (Fourth year and over) who have always suffered from the lack of practical skills. However, all participants who were medical students were included, whereas non-medical students and responses with missing data were excluded.

We shared a link to an online self-administered questionnaire designed on Google Form through social media (Facebook®, Telegram®, Messenger®), and students who see the link could enter it and fulfill the questionnaire. The questionnaire was launched on 29-September-2021 and was withdrawn on 10-October-2021. It was published in medical students formal University groups, 1000 students interacted with the questionnaire and 745 completed it, thus the approximate response rate is 74.5%.

The sample size (n) was determined by Cochran’s Sample Size Formula with the assumption of 95% confidence level (Z = 1.96), e is the margin of error which is 5%, p is the (estimated) proportion of the population which has the attribute in question, and it equals 50% (or 0.5), and q is 1 – p.

$$n=\frac{Z^2 pq}{e^2}$$

The required Sample size (n) for this study, applying the previous formula, is 385.

### Questionnaire

We adapted our questionnaire (Additional files 1 and 2) based on questionnaires used in similar previous studies [3, 9, 10] as a point of reference for validity and added questions about some important ideas in our community such as the influence of religion and Society’s perception of PPE. It was initially prepared in English, translated into Arabic (the local language) by language experts, and re-translated into English to check consistency in the meaning of words and concepts (before gathering the study sample). A pilot study was conducted on 50 students, and the questionnaire was reviewed according to the primary statistical study. The reliability of this tool in measuring acceptability was assessed using Cronbach’s Alpha coefficient and showed an acceptable value of (0.77).

The final questionnaire (Additional files 1 and 2) is subdivided into many domains, starting with demographic information (age, gender, university, academic year, academic performance, financial status as a way to speculate about the surrounding environment). Previous experience of being examined by a supervisor was also recorded.

Afterwards, questions aim to assess students’ attitudes toward PPE and the factors affecting it. Using the 5-point-Likert scale (from 1: strongly disagree to 5: strongly agree), students could indicate their acceptability of practicing PPE, examining the same and opposite gender, being examined by the same and opposite gender, and the influence of the COVID-19 pandemic on their acceptance. The same scale was used to express their views on whether the conflict in Syria and its outcome on lack of learning means contributed to poor physical examination skills. Multiple Choice Questions were used to estimate: 1- Social and religious influence on their attitude, 2- What areas students would accept, 3- The acceptance of being examined by a supervisor, 4- Their attitude toward having a supervisor while practicing PPE.

### Data analysis

Variables were described using absolute (n) and relative (%) frequencies, median and percentile values. One way ANOVA was used to analyze whether the differences between different Universities in the acceptance of PPE are significant, also for financial status, Religion and society, and academic performance. Moreover, independent-samples T Test to analyze whether the difference between Genders (males and females) in the acceptance of PPE is significant.

For supporting PPE during COVID-19 pandemic, one way ANOVA also was used to analyze whether the differences between different Universities and different financial status are significant, and independent-samples T Test to analyze whether the difference between Genders (males and females) is significant. IBM SPSS Statistics 25 Software was used in statistical analysis.

### Ethics statement

The study was in compliance with the Declaration of Helsinki for research involving human subjects. The Ethical Committee approved this study in the Faculty of Medicine at Damascus University, Syria (390, 19-5-2022). All our methods were carried out in accordance with relevant guidelines and regulations. Informed consent obtained from all the participants included in the study. We explained the purpose of the study to each participant and the way to answer the questionnaire and it was all voluntary, no names were taken so we provided anonymous data collection.

## Results

A total number of 745 online surveys were collected. After excluding all participants who were not medical students and missing data, the final participants’ number was 657.

76.4% of participants were from Syrian public medical colleges and 6.5% from Syrian private medical colleges. Also, 59.3% of participants were females and 40.7% were males, other sample details and characteristics are shown in Table 1.

**Table 1:** Sample characteristics

Variables	Frequency	Percent
Gender		
Male	268	40.7
Female	390	59.3
Academic year by 2020/2021		
First-year	14	2.1
Second-year	46	7.0
Third-year	147	22.3
Fourth-year	171	26.0
Fifth-year	110	16.7
Sixth year	131	19.9
Graduated	38	5.8
College		
Damascus University (public)	318	48.3
Aleppo University (public)	102	15.5
Albaath University (public)	83	12.6
Tishreen University (public)	55	8.4
Hama University (public)	22	3.3
Tartous University (public)	17	2.6
Alfourat University (public)	3	0.5
Kalamoon University (private)	7	1.1
Syrian Private University (SPU) (private)	43	6.5
Alandalus Private University (private)	6	0.9
Alhawash Private University (private)	2	0.3
Financial status		
Very good	111	16.9
Good	493	74.9
Bad	54	8.2
Academic performance		
Excellent	83	12.6
Very good	310	47.1
Good	216	32.8
Average	36	5.5
Acceptable	13	2.0

### PPE acceptance rates and its associate factors

**Fig. 1:**
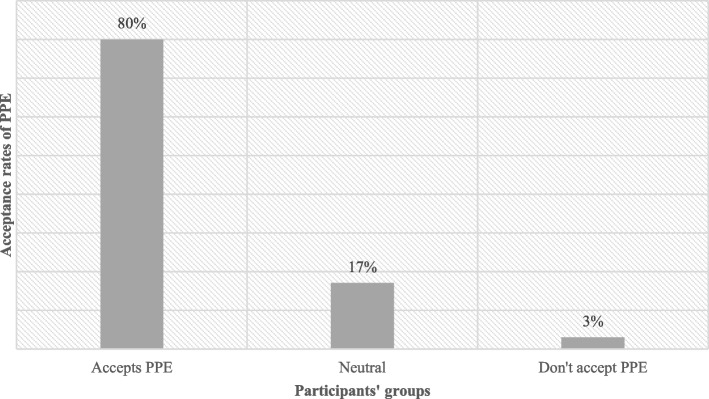
Acceptance rates of PPE among participants

Eighty percent of students accepted the Peer Physical Examination (PPE) (27% strongly agree, 56% agree); however, only 3% did not accept the PPE (0.3% strongly disagree, 2.7% disagree), and 17% were neutral (SD = 0.57) (Fig. 1).

**Fig. 2:**
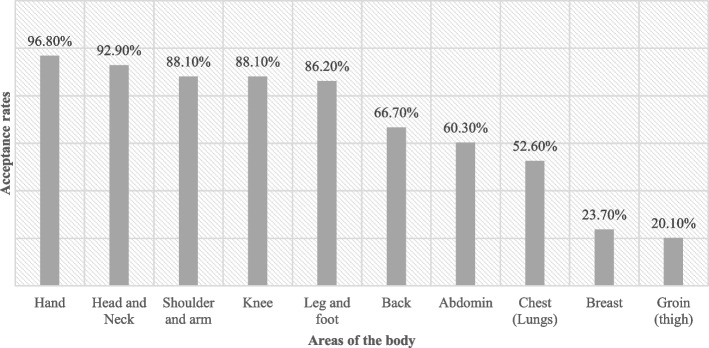
Acceptance rates for the areas of the body among participants

The groin area (thigh) was the most rejected area for examination (20% only accept it), followed by the breast (23%), then the chest (52%) and abdomen (60%), respectively, while the hand (96%) was the most acceptable area, followed by the head and neck (92%) (Fig. 2). Only 7.9% of females accepted the breast region to be examined while 46.6% of males accepted it and there is also a big difference in the abdominal region as only 44.3% of females accepted it while 83.5% of males accepted it, other regions and differences between males and females are shown in Table 2.

**Table 2:** Reported willingness to be examined on different body regions according to gender

Body region	Gender	
	Male	Female
Head and neck	94.4%	92.8%
Hand	96.2%	97.1%
Arm and shoulder	93.2%	84.6%
Breast	46.6%	7.9%
Chest (lungs and ribs)	81.7%	32.5%
Abdomen	83.5%	44.3%
Back	85.8%	53.5%
Groin	36.5%	8.7%
Leg and foot	90.6%	83.3%
knee	93.2%	84.6%

**Fig. 3:**
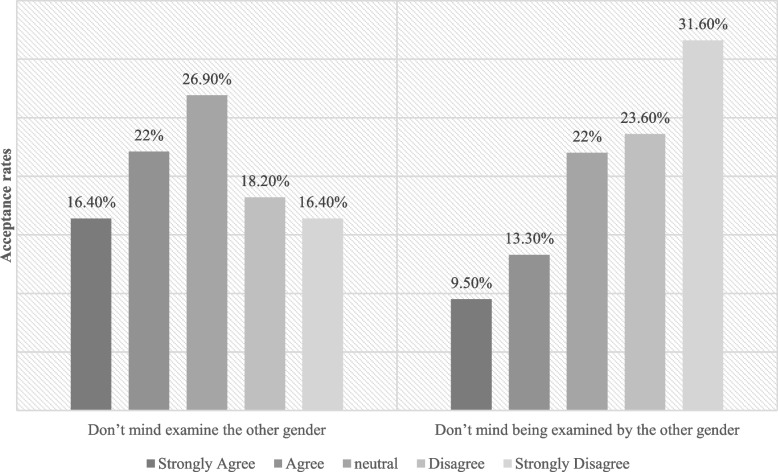
Females examination preference

**Fig. 4:**
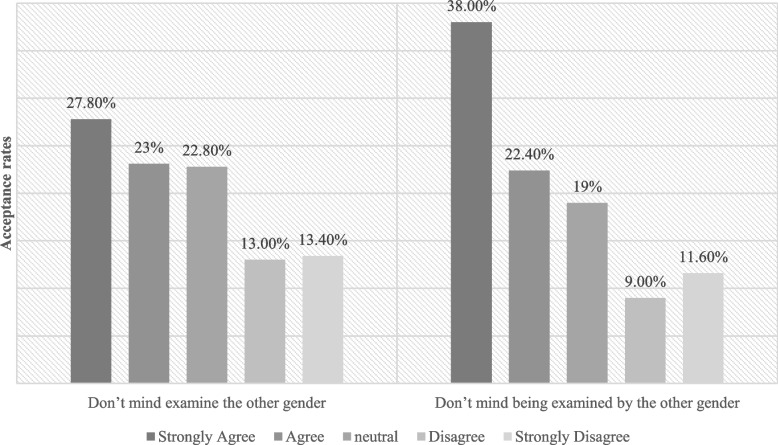
Males examination preference

This study found that male participants had statistically significantly higher acceptance rates of PPE (M = 3.94 out of 5) compared to female participants (M = 3.75), t(655) = 4.13, *p* < 0.001. Also, Participants who were previously examined by a supervisor had statistically significantly higher acceptance rates of PPE (M = 3.96) compared to those who were not examined (M = 3.73), t(655) = 5.26, *p* < 0.001. Females were less acceptable to being examined by the other gender than males (Figs. 3 and 4), 38% of females didn’t mind examining the other gender (50% for males), while only 22% didn’t mind being examined by the other gender (60% for males), these results were statistically significant with a *p*-value of (< 0.001).

A one-way ANOVA was performed to compare the effect of religion and society on acceptance of PPE, there was a statistically significant difference between groups as determined by one-way ANOVA (F(3,650) = 20.64, *p* < 0.001). A Tukey post hoc test revealed that the acceptance of PPE was statistically significantly lower in the group who stated that their religion believes is an obstacle towards PPE but their society accept it (M = 3.66, SD = 0.58, *p* < 0.001) and the group who stated that their religion believes is an obstacle towards PPE and their society as well (M = 3.58, SD = 0.52, *p* < 0.001) compared to the group who stated that their religion and society accept PPE (M = 3.97, SD = 0.56). There was no statistically significant difference between the group who stated that their society believes is an obstacle towards PPE but their religion accepts it and the group who stated that their religion and society accept PPE (*p* = 0.970). The obstacle of religion has affected females more than males (*p*- value = 0.002).

A one-way ANOVA was performed to compare the effect of financial status, University and academic performance on the acceptance of PPE. There was no statistically significant difference between having a very good financial status and good financial status and bad financial status (*p* = 0.249), Also There was no statistically significant difference between different Universities groups or between different academic performance groups with a *p*-value of 0.224 and 0.085, respectively.

### PPE acceptance during Covid-19 pandemic

**Fig. 5:**
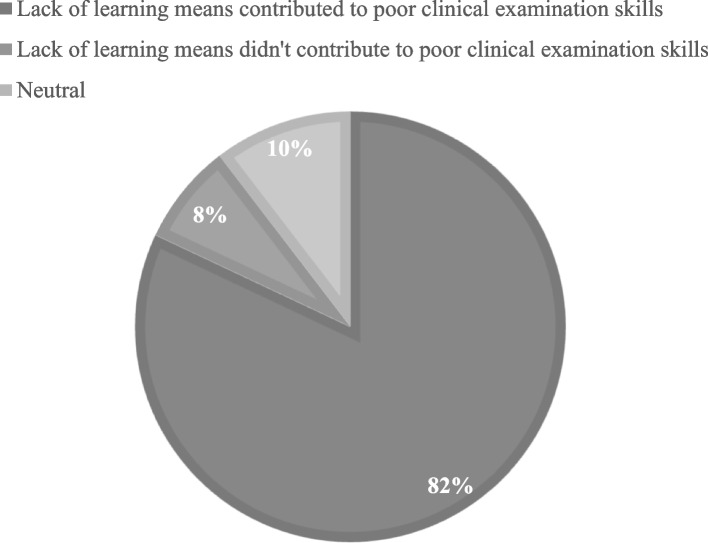
Relation between lack of learning means and poor clinical examination skills

Eighty-two percent of students saw that the lack of learning means because of the conflict contributed to poor clinical examination skills (61% strongly agree, 21% agree); however, only 7.6% did not see that (3% strongly disagree, 4.6% 12), and 10.4% were neutral (Fig. 5). Also, there was no statistically significant difference between different Universities groups with a *p*-value of 0.606.

**Fig. 6:**
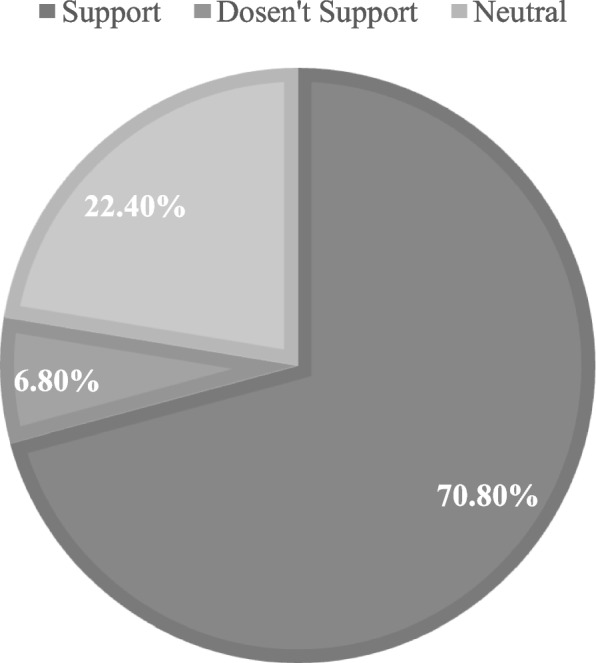
Supporting PPE during COVID-19 pandemic rates among participants

70.8% of students supported PPE during the COVID-19 pandemic and didn’t see Covid-19 pandemic as an obstacle at all (61% strongly agree, 21% agree); however, only 6.8% did not support PPE during the Covid-19 pandemic (2.4% strongly disagree, 4.4% disagree), and 22.4% were neutral (Fig. 6).

A one-way ANOVA was performed to compare the effect of financial status and University on the acceptance and support of PPE during the COVID-19 pandemic. There was no statistically significant difference between having a very good financial status group and good financial status group and bad financial status group (*p* = 0.290), Also There was no statistically significant difference between deferent Universities groups with a *p*-value of 0.128. A two-sample t-test was performed to compare acceptance and supporting of PPE during the COVID-19 pandemic between males and females. However, there was not a significant difference in acceptance and supporting of PPE during the COVID-19 pandemic between males and females, *p* = 0.267.

### Acceptance of being examined by a supervisor

77.7% of students accepted being examined by a supervisor; however, only 22.3% did not accept being examined by a supervisor. Males (90.7% of total males) accepted being examined by a supervisor more than females (71.8% of total females). These results were statistically significant, with a *p*-value < 0.001. Students attribute the reason for having a supervisor to point to mistakes and guide the student in the right direction (95.7%), feel more comfortable due to the Existence of an expert (78.9%), keeps it professional during examination (74%) and prevents disturbances and harassment (53%).

## Discussion

Peer Physical Examination PPE is becoming more and more crucial for medical schools to include in their curriculum, to allow their students to get more experience in clinical examination before dealing with real patients, the research aims to elucidate how all of these enigmatic demographic factors influence the willingness of Syrian students to participate in PPE taking into consideration the rough economic present state of the country and how that reflected upon the medical and educational equipment (life models) needed for their educational process. 82% of the medical students in our research agreed to the previous statement and confirmed its contribution to their poor clinical examination skills. Thus, PPE must be considered in to fill the gap in the medical educational system in Syria.

Unlike a similar study conducted in the UAE in which 47% of medical students would not want their peers to examine them as part of clinical skills learning, our survey showed that 75.7% of the students found PPE an appropriate way to build up their skills. That being said, doesn’t mean the actual application will go without any disruption because of the complicated structure the Syrian community has. Despite the students’ agreement to the importance of the PPE, their actual willingness to be engaged in the PPE is affected by their personal characteristics such as gender, religion, and ethnicity in addition to the social environment.

Our results (Fig. 1) showed a big difference between the willingness of the two genders to take part in the PPE; males were more willing to participate than females, which is considered to be an interesting result that points out many underlying reasons. Religion is probably the most controversial one as other studies found [11, 12], especially by the fact that the Syrian community is considered to be conservative, which limits the usage of PPE due to the possible interaction between the opposite genders which is forbidden in the Islamic faith. Even though the religious constraints affected both genders, our results indicated that females’ opinions were more related to religion than males’ opinions. This is constant to the results shown in previous studies about the relationship between gender and religion [13, 14]. Therefore, Females were more open to practice PPE within the same gender than across genders which is similar to other studies [9]. Actually, the same applies to males in our study but with lower refusal rates. This is consistent with the literature [7, 15] showing that single-gender pairing of students for PPE is more acceptable than across genders pairing. However, for students who were fine with mixed- gender groups, the survey results showed that females were more willing to take the Examiner role than being the examinee; on the contrary, male willingness rates were higher on the side of the Examinee role. That leads to male students being more disadvantaged in terms of opportunities to examine female peers, as reported in other studies [15, 16]. Additionally, Females have higher levels of body shame and body surveillance which contributes to them being less comfortable than males in the practice of PPE and probably creates a difference between the willingness of the two genders to participate in the PPE. They may also be less comfortable with mixed-gender PPE because they perceive males to be the perpetrators of critical and teasing comments and feel at risk of sexual objectification by males [2].

One of the most important factors that influence the efficacy of the PPE is the body areas that are accepted to be examined by the peer. It was expected that the students would be less comfortable with the intimate areas which were proven by our results; the groin area is the most unacceptable area to be examined among both males and females, followed by the breast. That agrees with the findings of [7, 11]. In contrast, the non- intimate areas were highly accepted to be examined by both genders which is similar to the findings of similar studies in western societies [3, 8]. However, there was a significant difference between females’ acceptance of the examination of specific parts of the body and males. These parts in our study were the abdominal and the back and since they are considered to be sensitive regions in the female body in the Syrian society and unacceptable parts to be exposed especially by the Muslims who constitute a huge part of the Syrian community, females had significantly lower percentages of acceptance to these parts’ examination. Whereas females in more conservative Arabian societies like UAE [10] were not comfortable with PPE of any part of their bodies.

Surprisingly, our results showed that there was no significant difference between students who agreed that society’s perception was an obstacle to the performance of PPE and students who agreed with the opposite on their acceptance of PPE. This is probably due to a small number of social differences among Syrian medical students like age and origin. While in the universities that have a greater diversity of students like Australia, its study showed an effect of being older and being from abroad on the PPE [17]. Although there is a good diversity in the financial status, academic performance, and diligence of Syrian medical students, the results showed that students who accepted to take the examinee role didn’t show signs of interest in the financial status of the fellow examiner nor his/her intelligence and diligence, We expected that smarter students would attract more students to choose them as their examiner, but it was proved wrong. This could be explained by the fact that all of the Syrian students lack practical experience and clinical skills so the examination might be the same if it was performed by any student regardless of his/her academic performance taking into consideration that the examination circumstances and hygiene must be the same in all of the PPE groups. However, the main factor that influenced them was choosing their examiner to be a friend or a stranger. Most of them favored having a friendly face probably because a friend could be more trustworthy than a stranger, there is less embarrassment between friends when it comes to body exposure and a friend is less likely to perform a painful examination. As a result, friendship could be used as a solution (by creating small groups of friends to practice with each other) for people who refused PPE due to shyness and embarrassment reasons; results showed that most students considered the role of examinee an embarrassing role in front of others, which may lead to bad experiences and even refusing to participate in PPE again. This embarrassment could also be explained by the lack of a safe environment during the session, most students who were examined by their tutor before showed more motivation to take part in PPE again, and that’s because of the safe and professional environment the presence of tutors can offer. Our results showed that 96% preferred having tutors around during sessions, and when asked about the importance of their presence, Students agreed on tutors` significant role in pointing out mistakes in students’ clinical examination and correcting them, as well as setting boundaries and preventing any kind of harassment. A previous study [18] showed that if there had been more tutors in the class, there would have been more participation.

At the end of the questionnaire, we asked the students if the current Covid-19 pandemic could be considered a problem for PPE, most of them didn’t find the pandemic to be any kind of barrier between them and practicing PPE, but of course, safety and protective measures must be taken and applied.

## Indications

Religious constraints should be respected, but clinical examination techniques must be learnt, religious strict students could join same-gender groups, thus groups shouldn’t be assigned by tutors, and freedom in pairing with others should be respected during PPE [18]. Our Results Showed that some students were more comfortable in friendship groups, while others chose to be with strangers, that leads us to the same important result, that there is no single successful strategy to follow which will promote willingness to participate. These findings underline the importance of choice in the composition of workgroups [5].

People who refuse to participate in PPE at all means could still learn through observing their colleagues taking turns in PPE; a previous study [19] provided a demonstration of the value of observation for the acquisition of clinical skills required to execute the physical examination.

## Limitations

Our research aimed to find whether there was a significant difference in the acceptance of PPE among students with different opinions towards the idea of religion, in general, being an obstacle to the performance of PPE. Thus, after we currently discovered their general connection, further research should ask the students about their specific religion (Muslim or Christian) and rate their level of religiousness, therefore making the results more precise to better understand the relation between religion and PPE.

## Conclusions

With an 80% acceptance rate, PPE represents an effective alternative to the absent life models in Syrian universities. But the more complicated societies get, the more challenges are in the means of learning.

This study identified that Religion and many other sensitive challenges (sensitive areas of the body, gender, safe environments, presence of tutors) are holding back the Syrian students from practicing PPE comfortably, building confidence, and taking their clinical skills to a higher level. While the definitive solutions are yet to be reached and achieved; some key ideas (such as the freedom of choosing your own PPE group) are available and can make big changes toward the real willingness to take part in PPE.

## Supplementary Information

**Additional file 1.**

**Additional file 2.**

## Data Availability

The datasets generated and/or analysed during the current study are not publicly available due it is in Arabic language and some restrictions apply to the availability of these data but are available from the corresponding author on reasonable request.

## Abbreviations

PPE: Peer Physical Examination
USA: United States of America
