# Supplementary material for: Syrian medical students’ acceptance of peer physical examination and its associating factors: a cross-sectional study
Source: BMC Med Educ. 2022 Dec 28;22:898. doi: 10.1186/s12909-022-03985-5 (PMC9795451; doi:10.1186/s12909-022-03985-5)
Supplement: Supplementary file 1 — Additional file 1. [file 12909_2022_3985_MOESM1_ESM.docx]

**
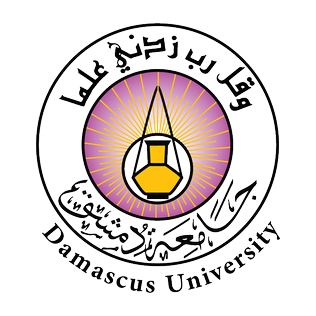
Questionnaire**

- Personal data:

Gender:

- Male.

- Female.

University year for 2020/2021:

- First-year.

- Second-year.

- Third-year.

- Fourth-year.

- Fifth-year.

- Sixth-year.

- Graduated.

University specialization:

- Faculty of Medicine.

- Faculty of Dentistry.

- Faculty of Pharmacy.

Other

The university you are currently studying at:

- Damascus University.

- Aleppo University.

- Al-Baath University.

- Tishreen University.

- Tartous University.

- Hama University.

- Al-Sham Private University.

- Syrian Private University (SPU).

- International University for Science and Technology (IUST).

- Kalamoon Private University.

- Al-Andalus Private University.

- Al-Hawash Private.

- Ittihad Private University (IPU).

- Arab International University (AIU).

What is your financial condition in general:

- Very good.

- Good.

- Bad.

Academic performance:

- Excellent
- Very good
- Good
- Moderate
- accepted
- Answer by choosing one of these 5 options:

|  | Strongly agree | Agree | Neutral | Disagree | Strongly disagree |
| --- | --- | --- | --- | --- | --- |
| It is imperative for a medical student to learn clinical examination skills during his time in the faculty |  |  |  |  |  |
| It is essential to practice clinical examination skills on healthy individuals before implanting these skills on patients (to discriminate between healthy and ill) |  |  |  |  |  |
| The shortage of practical learning means (like dummies) because of the Syrian crisis contributed to the decline of practical skills among medical students |  |  |  |  |  |

Have you ever undergone any of techniques of physical examination by your supervisor or a colleague for learning purpose?

Yes - No

Peer Physical Examination is a method of teaching and learning clinical skills where students act as model patients for one another to allow practice of physical examination techniques

Do you think peer physical examination could be adopted in our curriculum

- Answer by choosing one of these 5 options

|  | Strongly agree | Agree | Neutral | Disagree | Strongly disagree |
| --- | --- | --- | --- | --- | --- |
| In general, I think that practicing peer physical examination (PPE) is an appropriate way to acquire these skills |  |  |  |  |  |
| PPE can be used to learn the basic clinical skills but not every skill |  |  |  |  |  |
| The student might be embarrassed to perform or undergo PPE on his colleague |  |  |  |  |  |
| I feel comfortable when performing PPE on a colleague of my same sex |  |  |  |  |  |
| I feel comfortable when performing PPE on a colleague of my opposite sex |  |  |  |  |  |
| I feel comfortable when PPE is performed on me by a colleague of my same sex |  |  |  |  |  |
| I feel comfortable when PPE is performed on me by a colleague of my opposite sex |  |  |  |  |  |
| I feel more comfortable performing PPE on a friend |  |  |  |  |  |
| I feel more comfortable performing PPE on a colleague I don’t know |  |  |  |  |  |
| I don’t support PPE during covid-19 pandemic because it defies social distancing causing the disease to spread |  |  |  |  |  |
| We can practice PPE after taking measures of safety (vaccines – masks …) |  |  |  |  |  |
| During the pandemic, practicing PPE helps students learn the skills of disease spread prevention during clinical examination |  |  |  |  |  |
| I prefer that PPE is performed on me by my smarter colleague |  |  |  |  |  |
| I prefer that PPE is performed on me by academically superior colleague |  |  |  |  |  |

- I don’t mind being examined in:

1. Head and nick
2. Hand
3. Shoulder and arm
4. Breast
5. Chest (lungs and ribs)
6. Abdomen
7. Back
8. Inguinal area (palpation of femoral artery)
9. Leg and foot
10. Knee

- Based on your religious beliefs and your community customs and traditions, what is your opinion about performing PPE on a colleague of your opposite sex?

1. It doesn't interfere with my religious beliefs, But it's unacceptable in my community customs.
2. It doesn't interfere with my religious beliefs, and it's acceptable in my community customs.
3. It interferes with my religious beliefs, and it's unacceptable in my community customs.
4. It interferes with my religious beliefs, even though it's acceptable in my community customs

- If PPE is implemented, do you require a supervising professor or teacher?

Yes - No

- do you agree on being examined by a supervising teacher or professor?
- yes
- No
- what is importance of the presence of a supervising professor or teacher while practicing PPE (more than one choice can be picked)
- to warn the student if he makes a mistake
- to preserve the seriousness during the practice
- to avoid disturbance
- to make the examined more relaxed because of the presence of the experienced supervisor
- if you stand against PPE, do you prefer receiving the explanation and practicing on your family members?

Yes - No
