# Supplementary material for: Syrian medical students’ acceptance of peer physical examination and its associating factors: a cross-sectional study
Source: BMC Med Educ. 2022 Dec 28;22:898. doi: 10.1186/s12909-022-03985-5 (PMC9795451; doi:10.1186/s12909-022-03985-5)
Supplement: Supplementary file 2 — Additional file 2. [file 12909_2022_3985_MOESM2_ESM.docx]

**
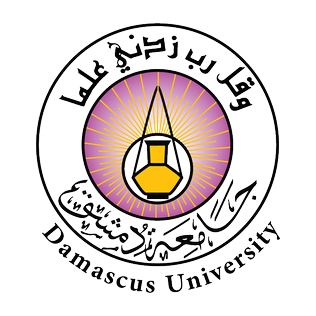
الاستبيان**

البيانات الشخصية:

الجنس:

- ذكر
- انثى

السنة الجامعية لعام 2020/2021

- السنة الأولى
- السنة الثانية
- السنة الثالثة
- السنة الرابعة
- السنة الخامسة
- السنة السادسة
- متخرج

التخصص الجامعي

- كلية الطب البشري
- كلية طب الاسنان
- كلية الصيدلة
- غير ذلك

الجامعة التي تدرس فيها حالياً

- جامعة دمشق
- جامعة حلب
- جامعة البعث
- جامعة تشرين
- جامعة طرطوس
- جامعة حماة
- جامعة الشام الخاصة
- الجامعة السورية الخاصة
- الجامعة الدولية للعلوم والتكنولوجيا
- جامعة القلمون الخاصة
- جامعة الاندلس الخاصة
- جامعة الحواش
- جامعة الاتحاد الخاصة
- الجامعة العربية الدولية

ماهي حالتك المادية بشكلٍ عام:

- جيدة جدا
- جيدة
- سيئة

مستواك الجامعي حسب معدلك:

- ممتاز
- جيد جدا
- جيد
- متوسط
- مقبول

أجب باختار أحد الخيارت الخمس التالية:

|  | أوافق بشدة | أوافق | محايد | لا أوافق | لا أوافق بشدة |
| --- | --- | --- | --- | --- | --- |
| 1. من الضروري لطالب الطب تعلم مهارات الفحص السريري خلال سنواته الدراسية |  |  |  |  |  |
| 1. يجب تطبيق مهارات الفحص السريري على أشخاص أسوياء لاكتساب هذه المهارات قبل تطبيقها على المرضى (تمييز السوي عن المرضي) |  |  |  |  |  |
| 1. ساهم نقص وسائل التطبيق العملي للفحص السريري (كالدمى) نتيجة ضعف الإمكانيات في ظل الأزمة السورية في تدني المهارات العملية لدى طلاب الطب |  |  |  |  |  |

هل سَبَقَ وأجرى أستاذك المشرف أو زميل لك إحدى تقنيات الفحص السريري عليك بهدف التعلم (جس- إصغاء أصوات القلب ...)؟

نعم – لا

الفحص السريري على الأقران Peer Physical Examination (PPE) هو طريقة لتعليم مهارات الفحص السريري يقوم فيها كل طالب بفحص زميله ثم يُفحص من قبل زميله

أي يؤدي الطالب دور المريض ليتم فحصه من قبل زميله بهدف اكتساب مهارات الفحص السريري

ما رأيك بتبني فكرة الفحص السريري على الأقران في مناهجنا العملية؟

أجب باختيار أحد الخيارات الخمس التالية:

|  | أوافق بشدة | أوافق | محايد | لا أوافق | لا أوافق بشدة |
| --- | --- | --- | --- | --- | --- |
| بشكل عام، أعتقد أنّ تطبيق الفحص السريري على الأقران طريقة مناسبة لاكتساب هذه المهارات |  |  |  |  |  |
| يمكن تطبيق الفحص السريري على الأقران للتعلم لكن ليس لكل المهارات |  |  |  |  |  |
| قد يشعر الطالب بالإحراج إذا فحص أو انفحص من قبل زميله |  |  |  |  |  |
| لا مانع لدي بإجراء الفحص( PPE ) على زميل من نفس الجنس |  |  |  |  |  |
| لا مانع لدي بإجراء الفحص ( PPE) على زميل من الجنس الآخر |  |  |  |  |  |
| لا مانع لدي بإجراء الفحص ( PPE) عليّ من قبل زميل من نفس الجنس |  |  |  |  |  |
| لا مانع لدي بإجراء الفحص ( PPE) عليّ من قبل زميل من الجنس الآخر |  |  |  |  |  |
| أشعر براحة أكبر إذا طبّقت الفحص على صدیقي |  |  |  |  |  |
| أشعر براحة أكبر إذا طبّقت الفحص على زمیل لا أعرفه |  |  |  |  |  |
| لا أدعم فكرة PPE خلال جائحة الكورونا كونها ستؤدي إلى تقارب يساعد على انتشار المرض |  |  |  |  |  |
| يمكننا تطبيق PPE مع اتخاذ إجراءات وقائية (لقاح- كمامة ..) |  |  |  |  |  |
| في ظل جائحة الكورونا تطبيق الفحص السريري على الاقران يساعد في تعلُّم اتخاذ وسائل الوقاية أثناء الفحص |  |  |  |  |  |
| أفضِّل أن يتم فحصي من زميلي الذي يبدو عليه الذكاء |  |  |  |  |  |
| أفضِّل أن يتم فحصي من زميلي الأكثر تفوقا دراسياً |  |  |  |  |  |

**لا أمانع في أن يتم فحصي في المناطق التالية (يمكن اختيار أكثر من خيار):**

1. الرأس والعنق
2. اليد
3. الكتف والذراع
4. الثدي
5. الصدر (الرئتين والأضلاع)
6. البطن
7. الظهر
8. الناحية المغبنية (مثل جس الشريان الفخذي ...)
9. الساق والقدم
10. الركبة

**وفقاً لمعتقداتك الدينية وعادات وتقاليد مجتمعك، ما هو موقفك من تطبيقك لل PPE على زميل من الجنس الآخر؟**

1- لا تتعارض مع معتقداتي الدينية، لكن الأمر غير مقبول ضمن عادات مجتمعي..

2- لا تتعارض مع معتقداتي الدينية، كما أن الأمر مقبول ضمن عادات مجتمعي..

3- تتعارض مع معتقداتي الدينية، إضافة لكون الأمر غير مقبول ضمن عادات مجتمعي..

4- تتعارض مع معتقداتي الدينية، على الرغم من كون الأمر مقبول ضمن عادات مجتمعي.

**في حال تطبيق الفحص السريري على الأقران هل تؤيّد وجود الدكتور المشرف (كفاحص/مشرف):**

- نعم
- لا

**هل توافق على أن يتم فحصك من قبل الدكتور المشرف:**

1. نعم
2. لا

**ما أهمية وجود الدكتور كمشرف عند تطبيق الفحص السريري على الأقران (يمكن اختيار أكثر من خيار):**

1. ينبه الطالب للخطأ في الفحص وتوجيهه بشكل صحيح
2. الحفاظ على الجديّة خلال التطبيق
3. تجنب المضايقات
4. اطمئنان المفحوص بشكل أكبر بسبب وجود خبير

**في حال لم تكنّ تؤيّد فكرة الفحص السريري على الأقران PPE هل تُفضِّل أن تتلقى الشرح ضمن جلسة العملي ثم تطبِّق ما تعلمته على أفراد أسرتك في المنزل؟**

1. نعم
2. لا
